# FcRider: a recombinant Fc nanoparticle with endogenous adjuvant activities for hybrid immunization

**DOI:** 10.1093/abt/tbae023

**Published:** 2024-09-06

**Authors:** Changchuin Mao, Karen Eberle, Xiaojie Chen, Yiming Zhou, Jun Li, Hong Xin, Wenda Gao

**Affiliations:** Antagen Pharmaceuticals, Inc., Canton, MA 02021, United States; Department of Microbiology and Immunology, Louisiana State University Health Science Center, New Orleans, LA 70112, United States; Base&Byte Biotechnology Co., Ltd., Changping District, Beijing 102206, PR China; Base&Byte Biotechnology Co., Ltd., Changping District, Beijing 102206, PR China; Department of Biological Sciences, Florida International University, Miami, FL 33199, United States; Department of Microbiology and Immunology, Louisiana State University Health Science Center, New Orleans, LA 70112, United States; Antagen Pharmaceuticals, Inc., Canton, MA 02021, United States

**Keywords:** adjuvant, vaccine, Fc, afucosylation, nanoparticle

## Abstract

Active immunization (vaccination) induces long-lasting immunity with memory, which takes weeks to months to develop. Passive immunization (transfer of neutralizing antibodies) provides immediate protection, yet with high cost and effects being comparatively short-lived. No currently approved adjuvants are compatible with formulations to combine active and passive immunizations, not to mention their huge disparities in administration routes and dosage. To solve this, we engineered the Fc fragment of human IgG1 into a hexamer nanoparticle and expressed its afucosylated form in Fut8−/− CHO cells, naming it “FcRider.” FcRider is highly soluble with long-term stability, easily produced at high levels equivalent to those of therapeutic antibodies, and is amenable to conventional antibody purification schemes. Most importantly, FcRider possesses endogenous adjuvant activities. Using SW_HEL_ B cell receptor (BCR) transgenic mice, we found that HEL-FcRider induced GL7^+^ germinal center B cells and HEL-specific IgG. Similarly, immunizing mice with UFO-BG-FcRider, a fusion containing the stabilized human immunodeficiency virus-1 (HIV-1) Env protein as immunogen, promoted somatic hypermutation and generation of long CDR3 of the IgG heavy chains. Intramuscular injection of (Fba + Met6)_3_-FcRider, a fusion with two peptide epitopes from *Candida albicans* cell surface, stimulated strong antigen-specific IgG titers. In three different models, we showed that afucosylated FcRider functions as a multivalent immunogen displayer and stimulates antigen-specific B cells without any exogenous adjuvant. As an antibody derivative, afucosylated FcRider could be a novel platform combining vaccines and therapeutic antibodies, integrating active and passive immunizations into single-modality “hybrid immunization” to provide complete and long-lasting protection against infections, and may open new avenues in cancer immunotherapy as well.

## Teaser

Statement of Significance: Active immunization (vaccination) and passive immunization (transfer of neutralizing antibodies) are both crucial for protection against pathogens, yet administered in mutually exclusive ways due to lack of compatible adjuvants. We glyco-engineered recombinant Fc nanoparticles, FcRider, with endogenous adjuvant activities and enhanced Fc-mediated effector functions to materialize the concept of “hybrid immunization.”

## Introduction

In immunology, an adjuvant is a substance that increases or modulates the immune response to a vaccine. Currently, only a few adjuvants other than aluminum salts (“alum”) have been licensed as components of vaccines in the USA. These are 4′-monophosphoryl lipid A (MPL), CpG oligonucleotides, MPL and QS-21 mixed in a liposomal formulation, and the oil-in-water emulsion MF59 [1]. While more are being developed in the research pipelines, these adjuvants have mechanisms of action (MOAs) that are either not well defined or mainly centered on various Toll-like receptor (TLR) pathways [2]. All these adjuvants/formulations are intended for intramuscular (i.m.) or subcutaneous (s.c.) injection, as they are often hydrophobic or oil-based, and show toxic side effects or could cause systemic inflammation and cytokine overproduction when applied directly through intravenous (i.v.) or mucosal routes.

These side effects, although taken for granted, actually limited our imagination in that immunization, the most genius way of humankind to fight infections, is forced to fall into two mutually exclusive camps: passive immunization (adoptive transfer of protective antibodies) and active immunization (vaccination). Oftentimes, in order to provide immediate protection, blocking or neutralizing antibodies are injected intravenously in formulated solutions without any adjuvant. For instance, the SARS-CoV-2-neutralizing antibodies REGN-COV2 and Sotrovimab are administered by intravenous infusion. On the other hand, vaccines are co-administered with adjuvants intramuscularly or subcutaneously with a delay in weeks or months for the appearance of induced highly potent antibodies and cellular responses. Can passive and active immunizations be combined in a single compatible mode, i.e. giving i.v. or i.m. injection of neutralizing antibodies while at the same time vaccinating the host, to provide more complete and long-lasting protection? For the first time, we propose the term “hybrid immunization” to describe this mode of immunization.

To probe for such a possibility, we focused on developing novel adjuvants that allow for co-administration of vaccines and therapeutic antibodies in a single modality. Just as TLRs expressed on various antigen-presenting cells (APCs), activating Fc gamma receptors (FcγRs) are also expressed on APCs, including monocytes/macrophages, granulocytes, dendritic cells, and follicular dendritic cells (FDCs) [3]. While TLR agonists are being exploited as adjuvants [1, 4], not much attention is drawn on engaging FcγRs for adjuvanticity. One major obstacle is that such FcγR-engagers have to effectively compete with endogenous IgG in vast molar excess in circulation or tissue, which is normally at around 10 mg/ml levels [5].

We hypothesize that if making a recombinant Ag-Fc fusion and using afucosylation technology to increase the binding of IgG Fc with activating FcγRs (human FcγRIIIA and mouse FcγRIV) [6], perhaps the Fc:FcγR interaction could transmit strong stimulatory signals to promote immunogen capture and presentation by APCs and functions as an intrinsic adjuvant. On the other hand, it is well known that Fc afucosylation enhances the effector functions of therapeutic antibodies by 50–100 fold [7], and the doses for their *in vivo* administration could be significantly reduced, making it possible to combine therapeutic antibodies and vaccines in a single entity. Out of this reasoning, we engineered an afucosylated (AF) two-unit tandem hIgG1 Fc joined by the trimerization sequence Foldon [8]. The murine version is similarly arranged as a two-unit tandem mIgG2a Fc joined by Foldon. We name these as “FcRider.” Herein, we report that FcRider is a hexamer nanoparticle. In three different models, recombinant Ag-FcRider expressed by Fut8−/− CHO cells (thus AF) possesses unique self-adjuvanting activities, which rely on both afucosylation and Fc oligomerization. As therapeutic antibodies can be easily engineered into the recombinant Ag-Fab-FcRider format, our findings pave the way for “hybrid immunization” that could fundamentally change the ways for humankind to fight against infections and other human diseases.

## Materials and methods

### Animals

Six- to ten-week-old female BALB/c mice and CD45.1 congenic mice on C57BL/6 background were purchased from the Jackson Laboratory (Bar Harbor, ME). SW_HEL_ BCR transgenic mice on C57BL/6 background were generated by Dr Robert Brink [9] and were imported from the laboratory of Dr James Zimring at University of Virginia. All experimental mice were kept in a temperature-controlled room with alternating 12-h dark–light cycles. Animal experimentation protocols were reviewed and approved by the Institutional Animal Care and Use Committees (IACUC) of Antagen Pharmaceuticals, Inc. and Louisiana State University Health Sciences Center’s IACUC (#4708).

### Reagents

AddaVax was purchased from InVivogen (San Diego, CA). Hen egg lysozyme (HEL), complete and incomplete Freund’s Adjuvant, MgCl_2_, L-arginine, L-glycine and all other chemicals were purchased from Sigma-Aldrich (St. Louis, MO). HyCell medium was a product of Cytiva (Marlborough, MA). All the molecular cloning enzymes and competent cells were from New England BioLabs (Ipswich, MA).

### Expression and purification of recombinant proteins

Genes coding for the human and mouse FcRider and related fusion proteins were constructed by overlapping PCR and cloned into pDirect4.2 CHO expression vector (Antagen, MA), sequencing verified (Azenta, NJ) and then electroporated (1620 V, pulse width 10 ms, 3 pulses) with the Neon™ electroporation system (Life Technologies, CA) into wild-type (WT) or Fut8−/− CHO-K1 cells (Antagen). One day after electroporation, cells were selected with Zeocin (300 μg/ml) in DMEM + 5% FBS for 2 weeks. Drug-resistant colonies were pooled together and transferred directly into HyCell serum-free medium (Cytiva) in shaking culture for 2–3 weeks. For Fc-containing proteins, culture supernatants were loaded onto Protein A columns (GenScript, NJ). After extensive washing with Phosphate-Buffered Saline (PBS), the bound proteins were eluted with 100 mM glycine-HCl (pH 2.5) in 10% sucrose and immediately neutralized with 1.5 M Tris.HCl (pH 8.5) to pH 7.0. For UFO-BG-FcRider, because the trimer structure of HIV-1 Env cannot withstand pH <4.5, the bound protein on Protein A column was eluted with mild elution buffer (MEB, 1.0 M MgCl_2_ and 1.0 M L-arginine at pH 5.0). For Hisx6-tagged proteins, culture supernatants were loaded onto Ni-INDIGO columns (Cube Biotech, Germany). After washing with a HEPES buffer containing 25 mM imidazole, the bound proteins were eluted with 150 mM imidazole in HEPES. Protein purity was examined by reducing and nonreducing SDS-PAGE (GenScript) and blue native PAGE (Life Technologies). BN-PAGE gels were run for 1.5 h at 150 V using the NativePAGE running buffer (Life Technologies), according to the manufacturer’s instructions. All the proteins were filtered through 0.22 μm before injection *in vivo*.

### Vaccination models and immunization

To measure antibody responses against HIV Env protein (UFO-BG) [10], 6–8-weeks-old female BALB/c mice (*n* = 3–4/group) were immunized on Weeks 0, 3, and 6 with injection (i.p.) of 50 μg UFO-BG-Hisx6 in PBS, or UFO-BG-Hisx6 in 1:1 volume mixture with AddaVax, or an equal molar amount of UFO-BG-mFcRider in PBS only. On Week 8, tail blood was harvested for enzyme-linked immunosorbent assay (ELISA) measurement of total serum IgG or IgG isotypes against UFO-BG.

To measure anti-HEL responses, 3 × 10^6^ splenocytes from SW_HEL_ BCR transgenic mice (CD45.2 in C57BL/6) were injected (i.v.) into CD45.1 congenic mice on Day −1. On Day 0, the recipients then received (i.p.) 50 μg HEL without any tag in PBS, or HEL emulsified with CFA (HEL + CFA), or the same molar doses of WT or AF HEL-mFcRider in PBS. The mice were boosted once on Day 9 or Day 12 in different experiments with the same antigens, except that CFA was replaced by IFA. On Days 22 and 24, tail blood was harvested and serum HEL–specific IgG and IgM levels were determined by ELISA.

To measure anti-*Candida* responses against the vaccine epitopes, female BALB/c mice were injected (i.m.) with 10 μg of AF (Fba + Met6)_3_-mFcRider or (Fba + Met6)_3_-mFcRider-CBP12 in PBS on Weeks 0, 3, and 6, and serum samples were taken 2 weeks after each immunization for ELISA.

### Enzyme-linked immunosorbent assay

To measure induced antibody titers against vaccine peptides, 96-well polystyrene plates were coated with synthetic peptides (Fba, Met6, purity >98.5%, GenScript) at 4 μg/ml in bicarbonate coating buffer and incubated overnight at 4°C. The following day, the wells were blocked with 1% BSA in PBS for 1 h at room temperature. Immune serum samples were then added in duplicate to respective wells using two-fold serial dilutions from 1:400 to 1:256 000 and incubated for 2 h at room temperature. Horseradish peroxidase (HRP)–conjugated goat anti-mouse polyvalent IgG (#12-349, Sigma-Aldrich) and IgM secondary antibody (#AP128P, Sigma-Aldrich) was then added (15000) and incubated in the dark for 1 h at room temperature. Tetramethyl benzidine (TMB) (#34022, Thermo Scientific, Waltham, MA) was then added to each well and incubated in the dark for 30 min at room temperature. HCl was added to stop the reaction, and the optical density was measured at 450 nm using a spectrophotometer. As a negative control, wells were incubated with secondary antibodies alone. Endpoint titers were given as the dilution whose optical density (OD) reading was greater than two times that of the negative control. Similar protocol was used to measure induced antibody titers against HIV Env or HEL, except that UFO-BG-Hisx6 or HEL was coated onto the plates at 2 μg/ml and blocked with DMEM + 5% FBS, and the immune sera were diluted in three-fold series. HRP-labeled anti-mouse IgG (#1030-05) or IgM (#1021-05) were purchased from Southern Biotech (Birmingham, AL).

### Flow cytometry analysis

Briefly, 0.5–1 × 10^6^ splenocytes in 100 μl aliquots were blocked with FcBlocker**™** and stained with FITC Annexin-V (#640906, BioLegend), Pacific Blue**™** anti-mCD45.2 (#109819, BioLegend), PE anti-IgM^b^ (#553521, BD Biosciences), and Alexa Fluor® 488 anti-GL7 (#144612, BioLegend) at 1.0 μg/ml on ice for 20 min. After washing with a Fluorescence Activated Cell Sorting (FACS) buffer (2% BSA in PBS), the cells were analyzed for fluorescence intensity on the BD LSRII instrument based on the manufacturer’s instructions.

### Immunoprofiling of bulk mouse BCR repertoire

After immunization with HEL-related antigens, mice were sacrificed and splenic B cells were enriched by negative selection with magnetic beads (Miltenyi Biotec). Total RNA was extracted from B cells of each mouse with the RNeasy Mini Kit (Qiagen). 5’-RACE and next-generation sequencing (NGS) for immunoprofiling the bulk BCR repertoire was outsourced to Azenta Life Sciences (Burlington, MA). BCR sequences were analyzed using the MiXCR tool v4.3.2 [11], and reference germline V, D, J, and C gene sequences were downloaded from the IMGT database [12].

### Statistical analysis

The data were analyzed using a paired Student *t* test and chi-square test, as appropriate.

## Results

### FcRider is a protein nanoparticle that functions as a multimeric immunogen displayer

To have higher effector functions of recombinant antibodies and Fc fusions, we deliberately enhance their engagement of the activating Fcγ receptors through: (i) afucosylation and (ii) Fc oligomerization. Afucosylation is achieved by expressing the proteins in Fut8−/− CHO cells. To have an oligomeric Fc, we engineered a two-unit tandem Fc joined by the trimerization sequence Foldon (Full sequence in Supplementary Information) and named it as FcRider.

**Figure 1:**
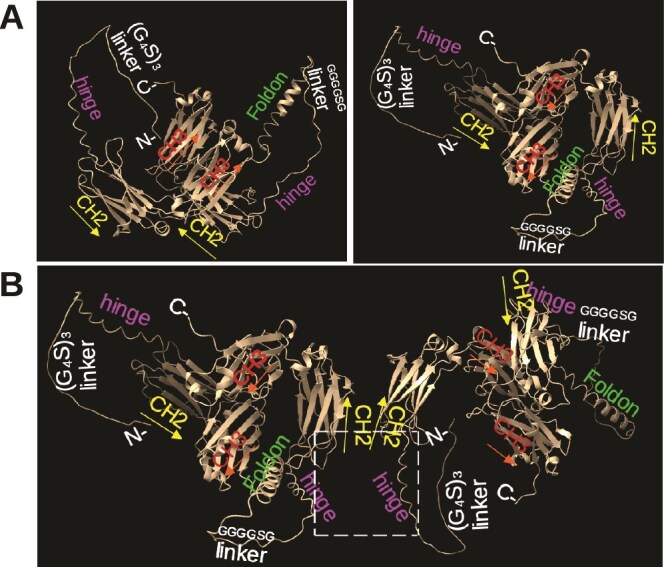
Model of human FcRider by ChimeraX software. (A) Snapshots of rotated FcRider 3D images. Domains are color-labeled. Note that the two CH3 domains of each Fc form a dimer within each FcRider. (B) Model of the two adjacent FcRider subunits within a possible hexamer. The dotted box indicates two hinges and upper CH2 regions necessary for asymmetric binding to hFcγRIIIA.

**Figure 2:**
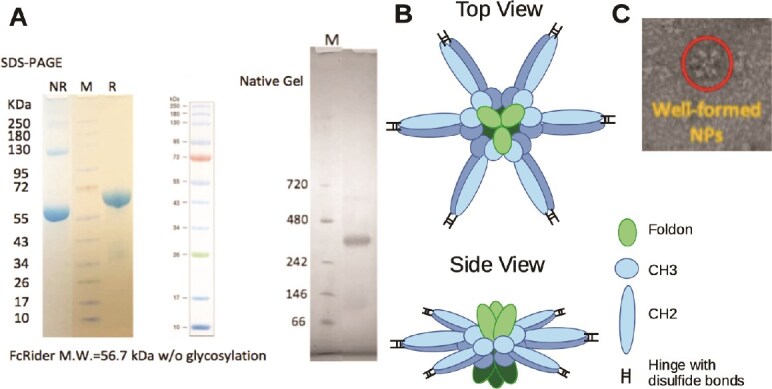
Model of human FcRider in hexamer organization. (A) Reducing (right, R) and nonreducing (left, NR) SDS-PAGE and native gel of hFcRider purified by Protein A column. (B) Top and side views of the FcRider hexamer in the “Lotus Model.” (C) Negative staining of UFO-BG-hFcRider in cryo-EM (more images are shown in Supplementary Fig. S2).

We used ChimeraX software to build the 3D structure of hFcRider. The model shows that the two CH2 domains within each FcRider molecule face opposite directions toward each other at a certain angle, and the two hinges of each FcRider molecule are even far apart (Fig. 1). Thus, they cannot form the two juxtaposed hinges and upper CH2 regions necessary for binding to FcγRs. Yet, we found that AF FcRider exhibits superior binding and strong activation of hFcγRIIIA (Supplementary Fig. S1). We propose that the necessary structure for binding to FcγRs is formed between two FcRider molecules (Fig. 1B, dotted box). Multiple pairs of disulfide bonds between the neighboring hinges may be formed, like in an IgG1 molecule, stabilizing the inter-FcRider interactions. Three sets of such dual-FcRider pair are further trimerized together by the three Foldon sequences on one side of the vertical axis and by another three Foldon sequences on the other side of the axis (Fig. 1). Note that the two green-colored Foldon sequences are each within a trimerized complex, with the trimer apex facing toward or away from the viewer, respectively. Thus, intra-FcRider CH3-CH3 interactions and inter-FcRider hinge-CH2 disulfide bond formation bring the whole quaternary FcRider structure into a hexamer, which is supported by M.W. analysis in SDS-PAGE and native gel (Fig. 2A).

Under reducing conditions, human FcRider is ~65 kDa in SDS-PAGE. Under nonreducing conditions, hFcRider is somewhat more compact and runs at ~60–62 kDa. The faint bands at 130 and 240 kDa are likely disulfide-bond linked 2 and 4 units of hFcRider. In native gel, hFcRider runs at ~360 kDa, indicating that it forms into a hexamer (Fig. 2A). We further developed a model to illustrate the organization of the six FcRider units in hexamer (Fig. 2B). Three units are joined together through the Foldon sequences on one plane, with the apex of the Foldon trimer facing vertically toward the viewer. Meanwhile, another three units similarly form a trimer on a parallel plane with the dark green–colored Foldon sequences facing vertically away from the viewer. The adjacent hinges and CH2 domains of each “petal” may form inter-FcRider disulfide bonds to stabilize the whole quaternary structure. We call this the “Lotus Model,” as it looks like a lotus flower floating on the placid surface of water. Negative staining images of hFcRider in cryo-EM indicate that hFcRider forms into nanoparticles with ~10 nm in size (Supplementary Fig. S2). We also generated a recombinant HIV vaccine with the uncleaved prefusion–optimized (UFO) envelope protein of the BG505 strain UFO-BG [10] fused at the N-terminus of hFcRider. The negative staining images of UFO-BG-hFcRider in cryo-EM indicate well-formed nanoparticles with ~20 nm in size (Fig. 2C, Supplementary Fig. S2).

### FcRider has intrinsic adjuvant activities different from that of the conventional adjuvant AddaVax

**Figure 3:**
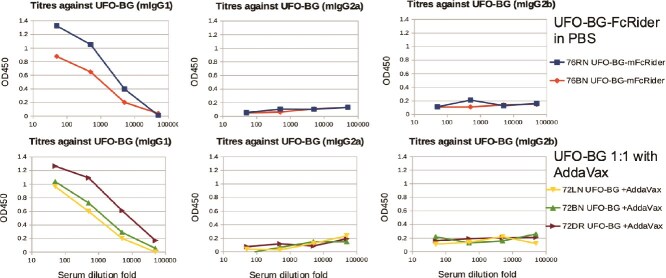
FcRider has intrinsic adjuvant activities. Immunization of BALB/c mice with UFO-BG-mFcRider AF in PBS in the absence of exogenous adjuvant induced similar mIgG titers and isotype patterns as those induced by UFO-BG in 1:1 volume mixture with the exogenous adjuvant AddaVax. Mice were immunized (i.p.) with 50 μg/mouse UFO-BG or the same molar amount of UFO-BG-mFcRider on Weeks 0, 3, and 6, and sera were collected on Week 8. Coated Ag in ELISA: UFO-BG-Hisx6. Number and letter combinations mark each individual mouse. Representative ELISA data were from one of the two similar experiments.

As a proof of concept for FcRider-based immunogen having intrinsic adjuvant activity, we immunized BALB/c mice with AF UFO-BG-mFcRider in PBS or with UFO-BG mixed with AddaVax on Weeks 0, 3, and 6. We found that AF UFO-BG-mFcRider could induce sufficient titers against coated UFO-BG-Hisx6 in ELISA, in a fashion virtually equivalent with UFO-BG + AddaVax. Both immunizations induced mIgG1 titers, but no mIgG2a or mIgG2b titers at Week 8 (Fig. 3). Under this condition, immunization with UFO-BG-Hisx6 alone in PBS without an adjuvant did not induce any antibody titers (not shown), as expected.

Although the titers were similar, we wanted to probe for any qualitative differences in Ig gene usage and somatic hypermutation (SHM), so we MACS-enriched the splenic B cells for immunoprofiling. Splenic B cells of three individual mice, each from the UFO-BG group (mouse #44LN), UFO-BG + AddaVax group (mouse #72LN), and UFO-BG-mFcRider AF group (mouse #76DR), were prepared, and total RNA was extracted for immunoprofiling through NGS.

**Figure 4:**
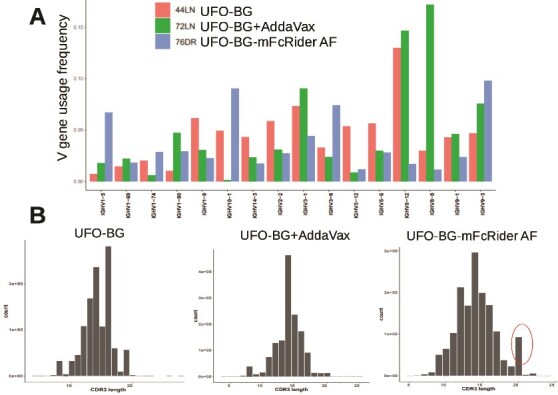
FcRider has adjuvant activities different from AddaVax and promotes SHM and long CDR3s. Heavy-chain germline gene usage (A) and distribution pattern of CDR3 length (B) of the mouse antibody repertoire after vaccination with UFO-BG in PBS, UFO-BG in AddaVax, or UFO-BG-mFcRider AF in PBS. Splenic B cells were enriched with MACS for immunoprofiling by NGS. The percentage of each germline gene family is plotted as a histogram. Only the major V gene families with sum of gene usage frequency ≥5% are shown. Note that long CDR3s of 20 aa (circle) were seen in mice immunized with AF UFO-BG-mFcRider.

First, the VH germline gene usage is different among the three groups (Fig. 4A). While vaccination with UFO-BG + AddaVax differentially expanded the usage of IGHV1-80, IGHV3-1, IGHV8-8, IGHV8-12 (*P* < 2 × 10^−16^, chi-square test) and suppressed the usage of IGHV10-1 and IGHV1-74 (*P* < 2 × 10^−16^), vaccination with UFO-BG-mFcRider AF differentially expanded the usage of IGHV1-5, IGHV3-6, IGHV10-1 (*P* < 2 × 10^−16^) and suppressed the usage of IGHV8-8 and IGHV8-12 (*P* < 2 × 10^−16^) (Supplementary Table 1). This means that although the induced antibody titers were similar, the repertoire of VH genes of anti-HIV antibodies could be substantially different.

Second, the complementarity-determining region 3 (CDR3) lengths of the heavy-chain VH genes were different among the three groups (Fig. 4B). Immunization with UFO-BG-mFcRider AF induced CDR3s of various lengths, more heterogeneous than those by UFO-BG + AddaVax. Most interestingly, antibodies with CDR3 length of 20 aa (Fig. 4B, red circle) distinctively appeared after immunization with UFO-BG-mFcRider AF (*P* < 2 × 10^−16^). NGS read counts for CDR3 length of 20 aa among the three groups are: 1372 (UFO-BG), 12 878 (UFO-BG + AddaVax), and 91 763 (UFO-BG-mFcRider AF) (Supplementary Table 1). Long heavy-chain CDR3 (HCDR3) loops are necessary for antibodies that neutralize microbial targets, including HIV. Several of the broadest and potently neutralizing HIV-specific antibodies have exceptionally long HCDR3s [13]. Our results suggest that FcRider-based immunogens induce more SHMs and favor the generation of long HCDR3s for potent neutralizing antibodies.

### FcRider induces GL7^+^ germinal center B cells, and this activity requires both Fc oligomerization and afucosylation

**Figure 5:**
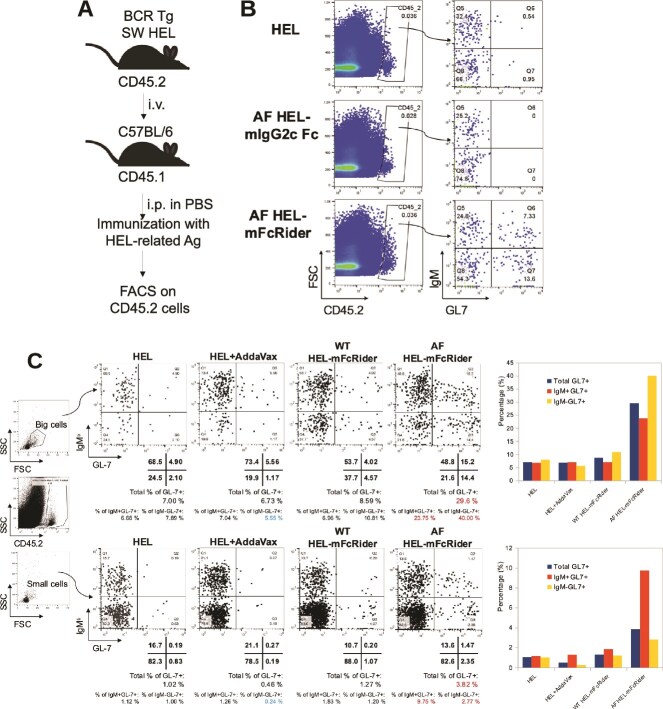
Oligomerization and afucosylation of Fc are necessary for FcRider’s adjuvanticity. (A) Illustration of immunization scheme. 3 × 10^6^ splenocytes from SW_HEL_ BCR transgenic (CD45.2 in C57BL/6) were injected (i.v.) into CD45.1 congenic mice on Day 0. The recipients then received (i.p.) 50 μg HEL without any tag in PBS or the same molar doses of AF HEL-mIgG2c or AF HEL-mFcRider in PBS on Days 1 and 9. On Day 13, splenocytes were prepared and Annexin-V-negative CD45.2^+^ cells were analyzed by FACS. Data representative of at least three similar experiments, with varying intervals between first and second immunizations. (B) AF HEL-mFcRider stimulated more GL7^+^ germinal center B cells than AF HEL-mIgG2c. (C) AF HEL-mFcRider is more potent than WT HEL-mFcRider in inducing GL7^+^ GC B cells. The cell adoptive transfer model was set up as in (A), and 1 day later, the recipients were immunized (i.p.) with 50 μg HEL, or with HEL in 1:1 mixture with AddaVax, or with WT or AF HEL-mFcRider in PBS. The amounts of the injected immunogens were normalized based on their molecular weights. Two weeks later, the mice were boosted again with the same antigens. After another 4 days, Annexin-V-negative CD45.2^+^GL7^+^IgM^b+^ GC B cells in the gated big cell population (upper panel) and small cell population (lower panel) were analyzed by FACS. The percentages of GL7^+^ cells after different immunizations were calculated based on quadrant percentages and plotted on the right.

To study the adjuvanticity of FcRider at single-cell levels, we used SW_HEL_ mice, a line of BCR transgenic whose B cells express BCRs carrying the specificity of the HyHEL10 monoclonal antibody (mAb) and bind the model protein hen egg lysozyme (HEL) [9]. WT and AF forms of HEL-mIgG2c and HEL-mFcRider were expressed from WT and Fut8−/− CHO cells respectively, and native HEL antigen without any tag was used as control. We injected (i.v.) 3 × 10^6^ splenocytes from SW_HEL_ mice (CD45.2^+^ C57BL/6) into the CD45.1 congenic strain of C57BL/6 mice. One day later, the recipients were immunized (i.p.) with HEL-based immunogens in PBS, with the protein amounts normalized against each immunogen’s M.W. After 9 days, the recipients were boosted (i.p.) with the same doses of immunogens. Four days later, mice were sacrificed, and splenocytes were analyzed by gating on Annexin-V-negative, CD45.2^+^ single cells (Fig. 5A). Strikingly, AF HEL-mFcRider stimulated a significant percentage (20.8%) of GL7^+^ germinal center (GC) B cells, among which 7.33% is IgM^+^ and the rest 13.6% is IgM^−^. Note that GL7 is a marker for GC B cells in the lymph nodes of immunized spleen, with GL7^high^-B cells being more functional in producing antibodies [14]. In contrast, HEL alone only stimulated 1.49% of GL7^+^ cells, whereas AF HEL-mIgG2c did not induce any at this time point (Fig. 5B). Similar results were obtained with another anti-HEL BCR transgenic line, MD4 (Supplementary Fig. S3). This indicates that Ag fused with one unit of IgG Fc is not sufficient to stimulate GL7^+^ GC B cells, and the oligomeric state of FcRider plays an important role for its intrinsic adjuvant activities.

Next, we tested whether (i) AF of FcRider contributed to the induction of GL7^+^ cells and (ii) how AF FcRider compared with conventional adjuvant AddaVax. In the same model, we found that 2 weeks after i.p. immunization in PBS, AF HEL-mFcRider stimulated significantly higher percentages of GL7^+^ GC B cells in both IgM^+^ and IgM^−^ compartments than WT HEL-mFcRider (3.4- and 3.7-fold respectively, Fig. 5C). This phenomenon is more dramatic in the gated population of large cells (more activated) than small cells (which also include non-B cells). At this early time point, AddaVax did not seem to induce more GL7^+^ cells than with antigen alone. It seems AddaVax just amplifies antigen stimulation, whereas Ag in fusion with FcRider, and particularly in the afucosylated form, delivers unique signals to trigger an earlier GC response. Our results indicate that both Fc oligomerization and afucosylation contribute to the induction of antigen-specific GL7^+^ GC B cells.

### FcRider is a vaccine carrier to induce strong IgG responses without exogenous adjuvant

**Figure 6:**
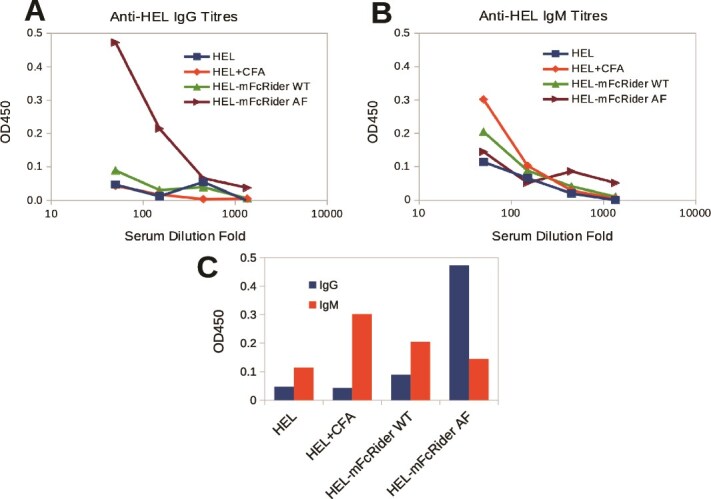
AF FcRider promotes class-switching and earlier IgG responses without exogenous adjuvant. 3 × 10^6^ SW_HEL_ splenocytes (CD45.2) were injected (i.v.) into CD45.1 congenic mice on Day 0. The recipients then received (i.p.) the same molar doses of HEL in PBS, HEL emulsified with CFA (HEL + CFA), WT HEL-mFcRider, or AF HEL-mFcRider in PBS on Day 1. The mice were boosted on Day 13, with the same antigens, except that CFA was replaced by IFA. On Days 23 and 25, sera were collected from tail veins and serum HEL-specific IgG (A) and IgM (B) titers were determined by ELISA, where plates were coated with 10 μg/ml HEL alone. AF HEL-mFcRider stimulated predominantly IgG responses over IgM responses. Note that AF FcRider is more potent than CFA in inducing IgG titers (C). Day 23 ELISA titers were presented. Day 25 ELISA titers were highly reproducible (not shown).

How about antibody responses in addition to the induction of GL7^+^ GC B cells? We showed that in the absence of any exogenous adjuvant, AF UFO-BG-FcRider can induce UFO-BG-specific IgG1 titers (Fig. 3). In the above adoptive transfer model using BCR transgenic donor B cells, we also found that AF HEL-mFcRider induced higher HEL-specific IgG titers than WT HEL-mFcRider without any exogenous adjuvant (Fig. 6A). It is quite surprising that the adjuvant effect of AF FcRider is even stronger than the classical adjuvant CFA/IFA, the latter of which only stimulated antigen-specific IgM response at this time point. Not only that, AF HEL-mFcRider promoted quick class-switching with an IgG-dominant response (Fig. 6B and C). Note that we did not clear the “space” of the recipients by irradiation or treatment with 5-fluorouracil (5FU), so after 3 weeks, there were very few donor SW_HEL_ mice–derived CD45.2^+^ cells that can be detected by FACS in the CD45.1 recipients. We assume that the adjuvant effect of AF FcRider would be more dramatic if the experiments were directly performed in SW_HEL_ mice.

**Figure 7:**
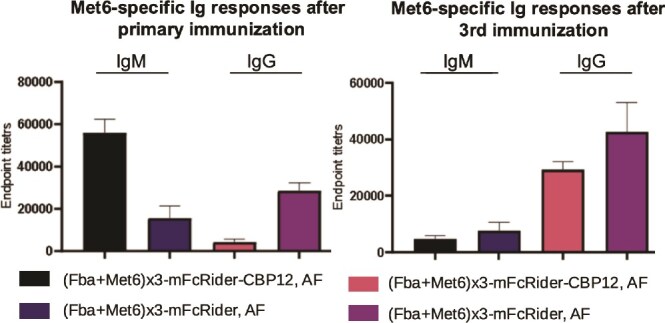
Intramuscular immunization with AF Ag-FcRider induced high IgG titers in ELISA. Synthetic Met6 peptide was coated on ELISA plates to measure specific IgM and IgG titers in sera after the primary and the third i.m. immunization with 10 μg/mouse of AF (Fba-Met6)_3_-mFcRider in BALB/c without any other adjuvant. CBP12 is a 12 a.a. peptide targeting to mouse CLEC9A on DC. Endpoint titers were given as the dilution whose OD reading was greater than two times that of the negative control. Data are presented as mean ± SD.

In a separate model, we constructed an experimental vaccine against *Candida albicans* by fusing Fba-Met6 epitopes [15] at the N-terminus of mFcRider and expressed it by Fut8−/− CHO cells. High titers of epitope-specific IgM and IgG could be detected after i.m. immunization of 10 μg of AF (Fba-Met6)_3_-mFcRider without any adjuvant in BALB/c mice. Primary immunization induced strong IgM response, whereas after the third immunization, IgG response became dominant (Fig. 7). As a side-by-side comparison, a similar construct with C-terminal addition of CBP12, a 12-aa peptide targeting CLEC9A on murine dendritic cells [16], seems to be less effective in inducing IgG responses but more prone to IgM induction, especially after primary immunization (Fig. 7). This suggests that FcRider may primarily engage an APC population other than the conventional dendritic cells (DC).

## Discussion

Global pandemics of infectious diseases call for novel strategies in building the immunity defense of the public. Passive and active immunizations both have advantages and disadvantages. Passive immunization can provide immediate protection, but only humoral immunity is transferred and the effects are relatively short-lived. Besides, the cost of substantial amounts of neutralizing antibodies, whether recombinantly expressed or harvested from convalescent sera, is not trivial. The high price tag could limit their wide administration in the general public. As an anecdote, former President Trump received 8 g of Regeneron’s SARS-CoV-2-neutralizing antibodies when he became positive for COVID-19 [17]. Obviously, ordinary people cannot afford such treatment. On the other hand, people taking immunosuppressive drugs (more than 6 million in the USA alone) and other immunocompromised populations, e.g. the elderly, are refractory or delayed in the induction of strong immunity even after multiple rounds of active immunizations (vaccination) [18]. Thus, a hybrid form of immunization combining the advantages of active and passive immunizations should be ideal in building immediate and long-lasting immunity against pathogens.

To our knowledge, in the real world, the closest situation to simultaneously administer neutralizing antibodies and vaccines is when giving postexposure prophylaxis (PEP) to prevent rabies. As rabies is virtually 100% fatal if not treated, human rabies immunoglobulin (HRIG) should be given as soon as possible to provide immediate protection before the vaccine-induced antibodies rise to sufficient levels. If possible, the full dose of the HRIG should be injected into and around the wound. HRIG and rabies vaccines should NEVER be given in or near the same place on the body [19], as the rabies virus envelope glycoprotein (RABV G) is the main component of the vaccine that HRIG reacts to. In addition to avoiding the canceling effect when the neutralizing antibodies react to the vaccine part, another important reason for physically separating active and passive immunizations is because of the lack of appropriate adjuvants. For co-administration to happen, such adjuvants should be potent enough but without toxicity and can be formulated even for i.m. or i.v. administration together with the neutralizing antibodies without adversely affecting their bioactivities.

In this study, we started to conceive the idea of “Hybrid Immunization,” first by engineering a novel adjuvant that can meet the above requirements. We obtained evidence from three different models that AF FcRider possesses endogenous adjuvant activities to stimulate antibody responses to the antigen that it carries. Intriguingly, both afucosylation and Fc oligomerization are necessary for such adjuvant activities. Understandably, due to much enhanced FcγRIIIA-binding affinity upon Fc defucosylation [20], Fc-containing proteins would be more easily captured by APCs expressing hFcγRIIIA (or its murine equivalent mFcγRIV) for antigen presentation, disregarding competition from low-binding normal plasma IgG even at ~10 mg/ml levels [5]. As an example, binding of fucosylated anti-CD20 to FcγRIIIA was almost abolished in the presence of human plasma and failed to recruit natural killer (NK) cells effectively, whereas AF anti-CD20 showed sufficiently high FcγRIIIA-binding activity to overcome competition from plasma IgG for binding to FcγRIIIA on NK cells [21]. Aside from this effect, crosslinking of the activating FcγRs by oligomerized Fc, i.e. FcRider in hexamer, could also activate FcγR^+^ APCs and transmit unique costimulatory signals for adjuvanticity. The two effects (higher binding and receptor crosslinking) could synergize to further amplify the FcγR-dependent adjuvant signal.

Structure-wise, we believe that the “Lotus Model” can explain why UFO-BG-hFcRider (and other Ag-FcRider as well) is highly expressed, highly soluble, and has long-term stability even at 4°C (UFO-BG-hFcRider was stored at 4°C for >6 months before cryo-EM). This may be because the CH3 dimers of each unit, together with the two Foldon cores, act like a molecular belt to restrain the hexamer. Overall, the nanoparticle hexamer structure inherits all the benefits of hIgG1 in terms of scalability and functionality, as well as easy purification by Protein A column. Due to its high valency, it can effectively present immunogen and crosslink activating FcγRs, and even more so when defucosylated, for enhanced FcγR-mediated adjuvant signal.

Unlike other nanoparticles that utilize bacterial or viral components (such as 24-mer *Helicobacter pylori* ferritin [22] or 60-mer *Aquifex aeolicus* lumazine synthase (LuS) [23]) for assembly, FcRider uses mainly human protein components except the 27-aa Foldon sequence, i.e. FcRider is a virtually human IgG1 Fc hexamer. The solubility, stability, scalability, and safety profiles of human IgG1 Fc have been proven in the antibody industry. The immunogenicity of FcRider is minimal, if any, compared to other nanoparticles composed entirely of foreign protein sequences. These foreign nanoparticles could elicit strong immune responses that: (i) deviate the protection focus away from the Ag part and (ii) preclude multiple administrations in the long term due to immunological memory to the foreign components of the nanoparticle carrier. If this first-generation FcRider does trigger antidrug antibodies (ADAs) *in vivo*, human trimerization sequences, such as those from human collagen and TNF/TNFR family members, can be used to replace Foldon in the construction of the next-generation FcRider.

Mechanistically, FcγRs recognize IgG-coated targets, such as opsonized pathogens or immune complexes (ICs), which are mimicked by recombinant fusion of Ag-FcRider nanoparticles. Crosslinking of activating FcγRs triggers downstream signaling cascades to activate FcγR-bearing APCs, leading to cargo internalization, antigen presentation, and T-cell stimulation [24]. Particularly, hFcγRIIIA is associated with immunoreceptor tyrosine-based activation motif (ITAM)-containing signaling molecules (FcRγ or CD3ζ chain homodimers or heterodimers), and this interaction is required for FcγRIIIA surface expression and signaling [25, 26]. Binding of ICs to the FcγRIIIA complex leads to Ca^2+^ influx, phosphatidylinositol 4,5 biphosphate hydrolysis, and an intracellular phosphorylation cascade [27, 28]. At present, it is not known which APC is involved in mediating FcRider’s adjuvant effect. Depending on the immunization routes, three types of APCs are primary candidates that may play central roles in FcRider-mediated adjuvanticity: tissue macrophages [29]; neutrophils [30, 31]; and FDCs.

In general, human and mouse macrophages express the activating hFcγRIIIA or mFcγRIV [3, 32] that is more sensitive to AF hIgG1 [6]. During infection, tissue macrophages serve as a bridge between innate and adaptive immunity [29]. For instance, spleen marginal metallophilic macrophages (MMMs) and marginal zone macrophages (MZMs) play important roles in capturing pathogens, ICs, and apoptotic materials and in cross-priming B, T, and dendritic cells, allowing for the downstream amplification of the immune response [33]. In addition, SIGN-R1^+^ MZMs are essential for the maturation of GC B cells in the spleen [34]. Upon entering the circulation, Ag-FcRider nanoparticles likely activate these small populations of splenic macrophage subsets, through which to promote GC response for the generation of high-affinity antibody-producing cells and memory B cells, as suggested by our studies (Figs 4 and 5).

On the other hand, neutrophils, also known as polymorphonuclear (PMN) leukocytes, are the most abundant leukocyte in human blood [35]. Mouse neutrophils express mFcγRIV [3], whereas human neutrophils express only low levels of FcγRIIIA, which are usually masked by the high levels of homologous FcγRIIIB [36]. Nonetheless, AF anti-CD20 activated neutrophils more efficiently than fucosylated anti-CD20 in the presence or absence of FcγRIIIB (which is a glycosylphosphatidylinositol (GPI)-anchored decoy receptor), indicating AF hIgG1 can activate neutrophils via FcγRIIIA [36], as other FcγRs (FcγRI and FcγRIIA/B) do not differentiate AF vs. fucosylated antibodies [20, 37]. Under acute inflammatory conditions, neutrophils rapidly migrate to not only sites of inflammation but also draining lymph nodes and spleen, where they bidirectionally engage with B- and T-cell subsets [38]. Unlike circulating neutrophils, B-cell helper neutrophils (NBh) residing in regional lymph nodes and in the marginal zone of the spleen readily release preformed Pentraxin 3 (PTX3) from secondary granules [39]. Binding of PTX3 to marginal zone B (MZB) cells promotes homeostatic production of IgM and class-switched IgG antibodies and expansion of plasmablasts [40]. PTX3 is thus considered to bridge the humoral arms of the innate and adaptive immune systems by serving as an endogenous adjuvant for innate-like B-cell subsets, including MZB cells and B-1 cells [40]. Our finding that immunization of SW_HEL_ B cells with AF HEL-mFcRider rapidly induced HEL-specific IgG, even more efficiently than with the conventional adjuvant CFA/IFA (Fig. 6), suggests that some endogenous adjuvant system may also be employed and the NBh-PTX3 axis could be a potential candidate.

The third cellular component possibly contributing to FcRider’s adjuvant activities is FDCs, which reside in primary follicles and in germinal centers of secondary and tertiary lymphoid organs [41]. They express Fc receptors and complement receptors that can capture and display Ag-Ab immune complexes, especially those particulate antigens [42], for months to years on their surface. FDCs play a crucial role in B-cell activation, SHM, and affinity maturation of antibodies [43]. Our findings that AF FcRider induces GL7^+^ GC B cells, promotes SHM with longer CDR3s in heavy-chain VHs, and stimulates class-switching and IgG secretion all hint that FDCs could also contribute to the adjuvanticity of FcRider. As a particulate nanoparticle, Ag-FcRider may be more easily captured by FDCs [42], displayed on their surface and presented to the developing B cells for a long time to incorporate SHM. This working hypothesis is supported by the findings that antigens displayed by nanoparticles can potently induce specific IgG antibodies at much reduced doses compared to the same antigens in soluble forms [22, 23, 42].

Regardless of potential mechanisms, because FcRider contains hinge and Fc sequences, it can be easily engineered to reconstitute with the Fab portion of a neutralizing antibody into the format of Ag-Fab-FcRider or alike (Supplementary Fig. S4). In this hybrid molecule, the Ag part can be the immunodominant substructures of a pathogen or short amino acid sequences containing characterized B- and T-cell epitopes, as long as they do not directly interact with the Fab portion of Ag-Fab-FcRider. If so, such a hybrid molecule is simultaneously both a neutralizing antibody and a vaccine. On one hand, when Fab is fused into Ag-FcRider, the resulting antibody oligomer with enhanced effector functions can provide passive immunization. On the other hand, when an immunogen is fused with Fab-FcRider, the resulting vaccine with endogenous adjuvant activity can impart active immunization.

There are ground-breaking implications for a successful self-adjuvanting Ag-Fab-FcRider. First, the conceived strategy of “Hybrid Immunization” now becomes possible. A recombinant neutralizing mAb-injected i.v. or i.m. targeting one particular antigen (Ag1) in the Ag2-Fab(anti-Ag1)-FcRider format can carry vaccine components of a different antigen (Ag2) or a different epitope of the same antigen. For example, two vulnerability sites of malaria infection have been identified at various stages of *Plasmodium falciparum* life cycle that are attractive targets for adoptive mAb therapy and for vaccine development. One is the junctional epitope of *P. falciparum* circumsporozoite protein (PfCSP) that is recognized by neutralizing mAbs MGG4, CIS43, L9, and others [44–46]. The other is mosquito midgut proteins and their cognate malarial binding partners involved in parasite transmission into the insect vector, which are targets of transmission-blocking vaccines (TBVs) [47, 48]. Giving AF TBV-antiPfCSP-FcRider not only can protect a person from sporozoite infection of hepatocytes but also will at the same time immunize the host to generate antibodies to thwart parasite transmission into mosquitoes. This hybrid immunization strategy at community level could be more effective than targeting either of the two vulnerability sites separately to interrupt the life cycle of *P. falciparum*. It could be envisioned that hybrid immunization is a paradigm shift in basic immunology, and it will catalyze a whole new area of research in infectious diseases, vaccine development, and cancer immunotherapy. For the latter, combining conventional therapeutic antibodies with cancer vaccines would greatly expand our armamentarium to treat cancers and could substantiate the AntibodyPlus concept [49] first proposed by the Chinese Antibody Society (CAS).

Second, because the self-adjuvanting Ag-Fab-FcRider can be recombinantly expressed and exert its effects without any exogenous adjuvant, which is often toxic or highly inflammatory, this opens up new possibilities of “edible biologics” and “oral vaccines” [50]. For example, microalgae species, such as *Chlamydomonas reinhardtii*, *Chlorella protothecoides*, and *Haematococcus pluvialis*, have been granted GRAS (Generally Regarded As Safe) status by the Food and Drug Administration (FDA) and are edible with high nutraceutical values for humans or could be used as feeds for animals [51, 52]. The gene coding for Ag-Fab-FcRider can be inserted into the chloroplast genome of the green algae and stably expressed to high levels inside this organelle [53]. The algal expression system can produce highly complicated proteins with disulfide bonds, such as enzymes and full antibodies, including recombinant immunotoxins for cancer therapy [54, 55]. Once the biomass of recombinant microalgae is lyophilized into dry powder, the bioactivities of biologics, including vaccines, can be retained for at least 1.5–2 years at room temperature [56]. Orally taken microalgae tablets serve as a delivery vehicle for bioencapsulated antibodies and vaccines to protect them from denaturing by low pH and degradation by proteases in the harsh environment of the stomach. Only in the small intestines, the cell wall of microalgae is degraded by enzymes from the gut-residing microbes to release the cargo for absorption into the circulation via FcRn-mediated transcytosis, if the cargo is an Fc-containing biologic. This process completely obviates the costly steps of mammalian cell culture, protein purification, cold chain storage, and needle injection and will fundamentally change the landscape of healthcare [53]. Not only microalgae, other organisms of GRAS status, such as *Lactobacillus*, can be engineered to express Ag-Fab-FcRider for oral delivery of mucosal vaccines [57, 58] or for hybrid immunization. Via the oral route, not only systemic immunity but also mucosal immunity can be induced to block infections at the very first line of defense, i.e. mucosa, which is the entry port for most infectious agents. This novel mode of oral hybrid immunization can also be extended to agricultural animals, if they are fed with Ag-Fab-FcRider expressing microalgae, to prevent infections.

In summary, we for the first time described an engineered Fc nanoparticle that exhibits endogenous adjuvant activities to potentially enable combining active and passive immunizations into hybrid immunization. Although the bioavailability, efficacy, and safety profiles of Ag-Fab-FcRider need to be tested in future studies, the endless possibilities brought up by this novel concept would catalyze the generation of new medicine and revolutionize the way of health management.

## Supplementary Material

Supplementary_Materials_tbae023

## Data Availability

The data underlying this article are available in the article and in its online supplementary material.

## Animal research statement

Animal experiment protocols were reviewed and approved by the Institutional Animal Care and Use Committees (IACUC) of Antagen Pharmaceuticals, Inc. (OLAW assurance #D22–01144), and that of Louisiana State University Health Sciences Center’s IACUC (#4708).
