# Supplementary material for: FcRider: a recombinant Fc nanoparticle with endogenous adjuvant activities for hybrid immunization
Source: Antib Ther. 2024 Sep 6;7(4):295–306. doi: 10.1093/abt/tbae023 (PMC11456856; doi:10.1093/abt/tbae023)

**FcRider: A Recombinant Fc Nanoparticle with Endogenous Adjuvant Activities for Hybrid Immunization**

**Supplementary Materials**

Human FcRider:

**Signal**_linker_hIgG1Fc_linker_Foldon_linker_hIgG1Fc

**MWWRLWWLLLLLLLLWPMVWA**SSLEGGGGSGGGGSGGGGSPKSCDKTYTCPPCPAPELLGGPSVFLFPPKPKDTLMISRTPEVTCVVVDVSHEDPEVKFNWYVDGVEVHNAKTKPREEQYNSTYRVVSVLTVLHQDWLNGKEYKCKVSNKALPAPIEKTISKAKGQPREPQVYTLPPSRDELTKNQVSLTCLVKGFYPSDIAVEWESNGQPENNYKTTPPVLDSDGSFFLYSKLTVDKSRWQQGNVFSCSVMHEALHNHYTQKSLSLSPGGSGYIPEAPRDGQAYVRKDGEWVLLSTFLGGGGSGPKSCDKTYTCPPCPAPELLGGPSVFLFPPKPKDTLMISRTPEVTCVVVDVSHEDPEVKFNWYVDGVEVHNAKTKPREEQYNSTYRVVSVLTVLHQDWLNGKEYKCKVSNKALPAPIEKTISKAKGQPREPQVYTLPPSRDELTKNQVSLTCLVKGFYPSDIAVEWESNGQPENNYKTTPPVLDSDGSFFLYSKLTVDKSRWQQGNVFSCSVMHEALHNHYTQKSLSLSPGK

GOI coding for Ag or Fab can be inserted at NheI (gctagc, AS) and XhoI (ctcgag, LE) sites.

Mouse FcRider:

**Signal**_linker_mIgG2aFc_linker_Foldon_linker_mIgG2aFc

**MWWRLWWLLLLLLLLWPMVWA**SSLEGGGGSGGGGSGGGGSPRGPTIKPCPPCKCPAPNLLGGPSVFIFPPKIKDVLMISLSPIVTCVVVDVSEDDPDVQISWFVNNVEVHTAQTQTHREDYNSTLRVVSALPIQHQDWMSGKEFKCKVNNKDLPAPIERTISKPKGSVRAPQVYVLPPPEEEMTKKQVTLTCMVTDFMPEDIYVEWTNNGKTELNYKNTEPVLDSDGSYFMYSKLRVEKKNWVERNSYSCSVVHEGLHNHHTTKSFSRTPGGSGYIPEAPRDGQAYVRKDGEWVLLSTFLGGGGSGPRGPTIKPCPPCKCPAPNLLGGPSVFIFPPKIKDVLMISLSPIVTCVVVDVSEDDPDVQISWFVNNVEVHTAQTQTHREDYNSTLRVVSALPIQHQDWMSGKEFKCKVNNKDLPAPIERTISKPKGSVRAPQVYVLPPPEEEMTKKQVTLTCMVTDFMPEDIYVEWTNNGKTELNYKNTEPVLDSDGSYFMYSKLRVEKKNWVERNSYSCSVVHEGLHNHHTTKSFSRTPGK

GOI coding for Ag or Fab can be inserted at NheI (gctagc, AS) and XhoI (ctcgag, LE) sites.

**
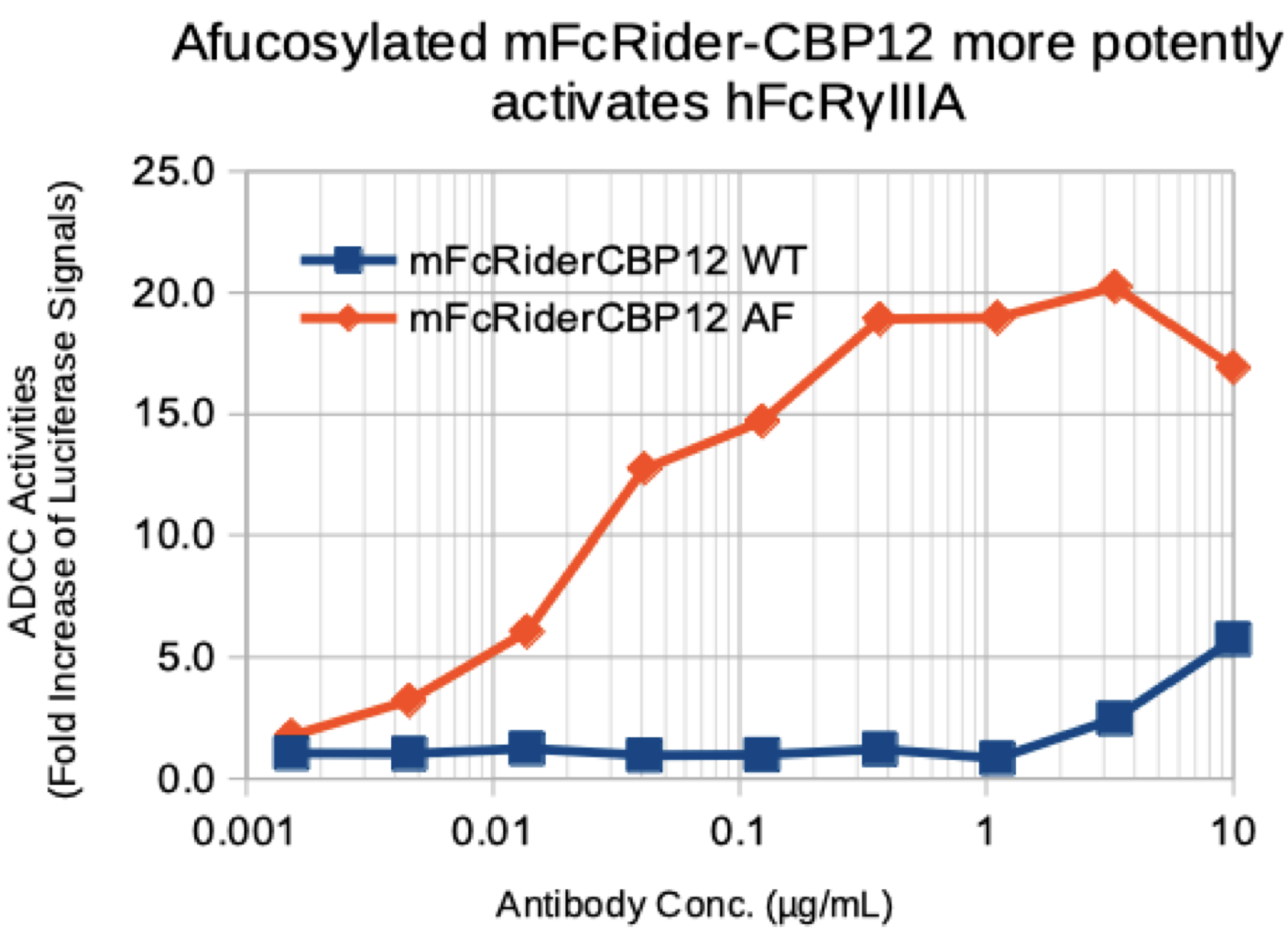
**

**Supplementary Fig. S1.** Afucosylated and oligomerized Fc can strongly engage and crosslink the activating FcγR. Engineered Jurkat T cells expressing hFcγRIIIA and FcRγ were incubated with titrating doses of WT or AF mFcRiderCBP12, produced by WT or Fut8-/- CHO cells. hFcγRIIIA crosslinking and activation trigger downstream signaling events that drive the expression of luciferase, in a reporter assay to measure ADCC activities. Similar findings were also obtained if AF and WT hFcRider were used, whereas adding regular mIgG2a or hIgG1 alone did not induce any luciferase signals (not shown).


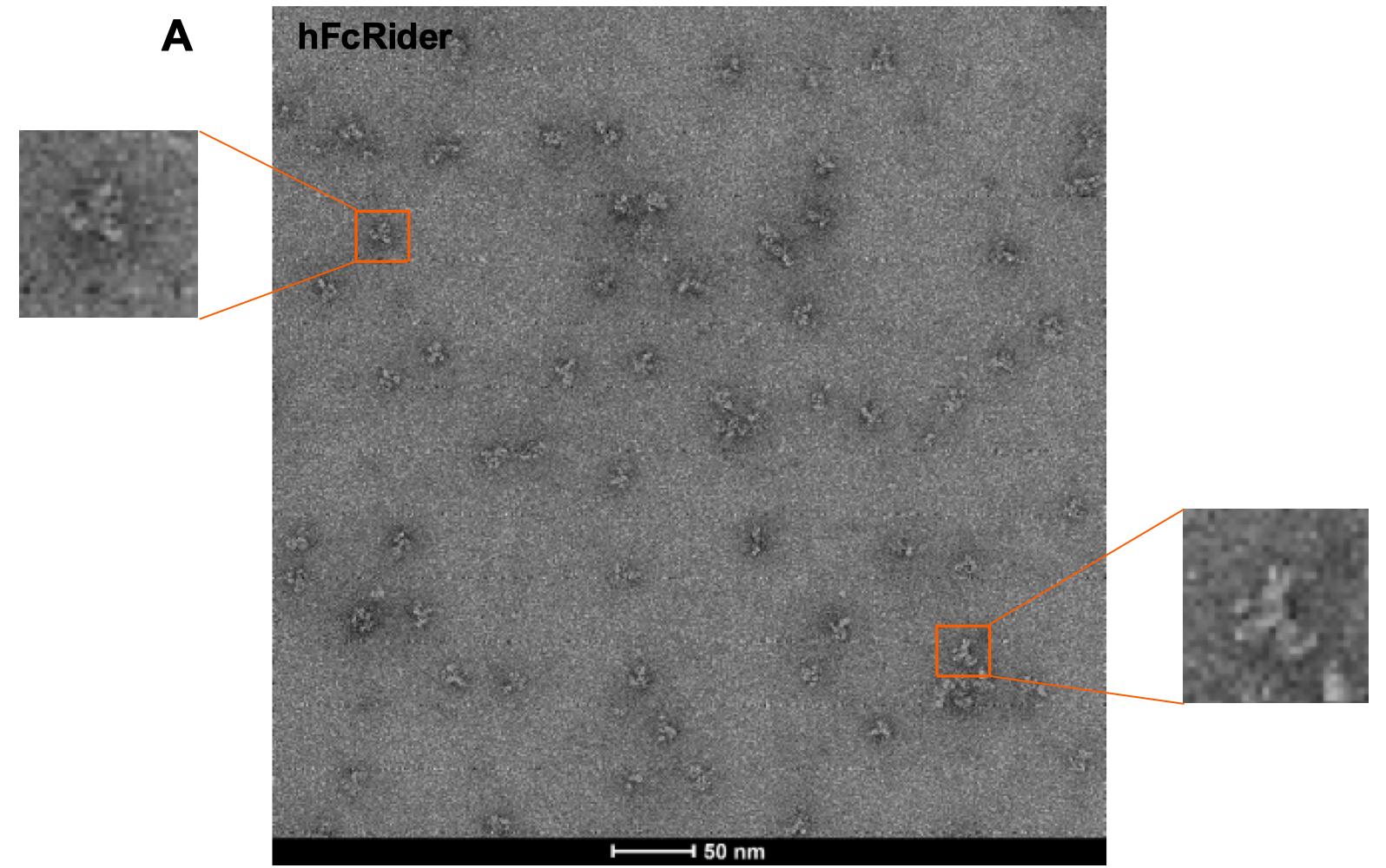


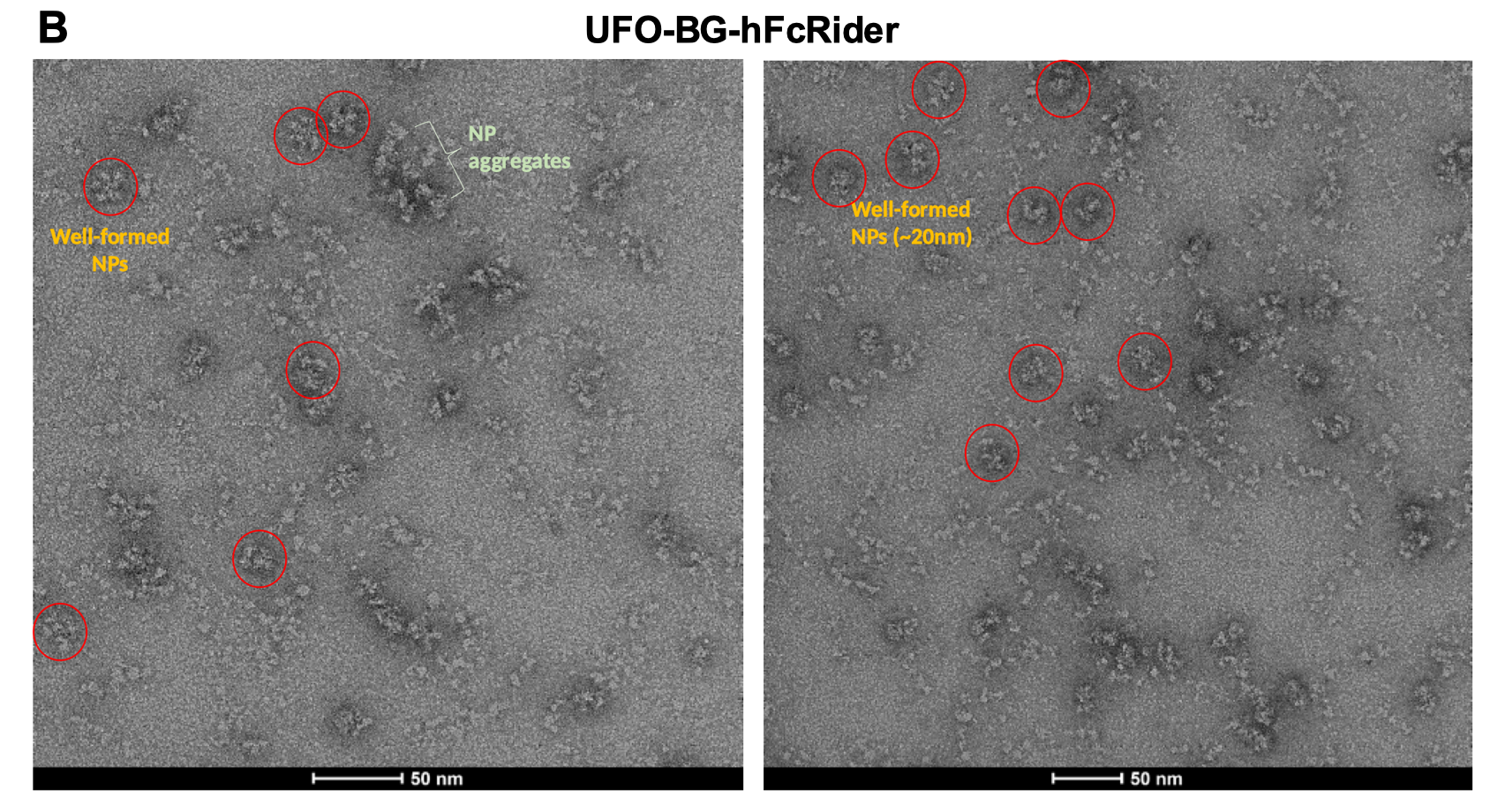
**Supplementary Fig. S2.** Negative staining images of hFcRider (A) and UFO-BG-hFcRider (B) in cryo-EM. Bar size is 50 nm. FcRider is about 10 nm in diameter, while UFO-BG-hFcRider is about 20 nm in diameter.


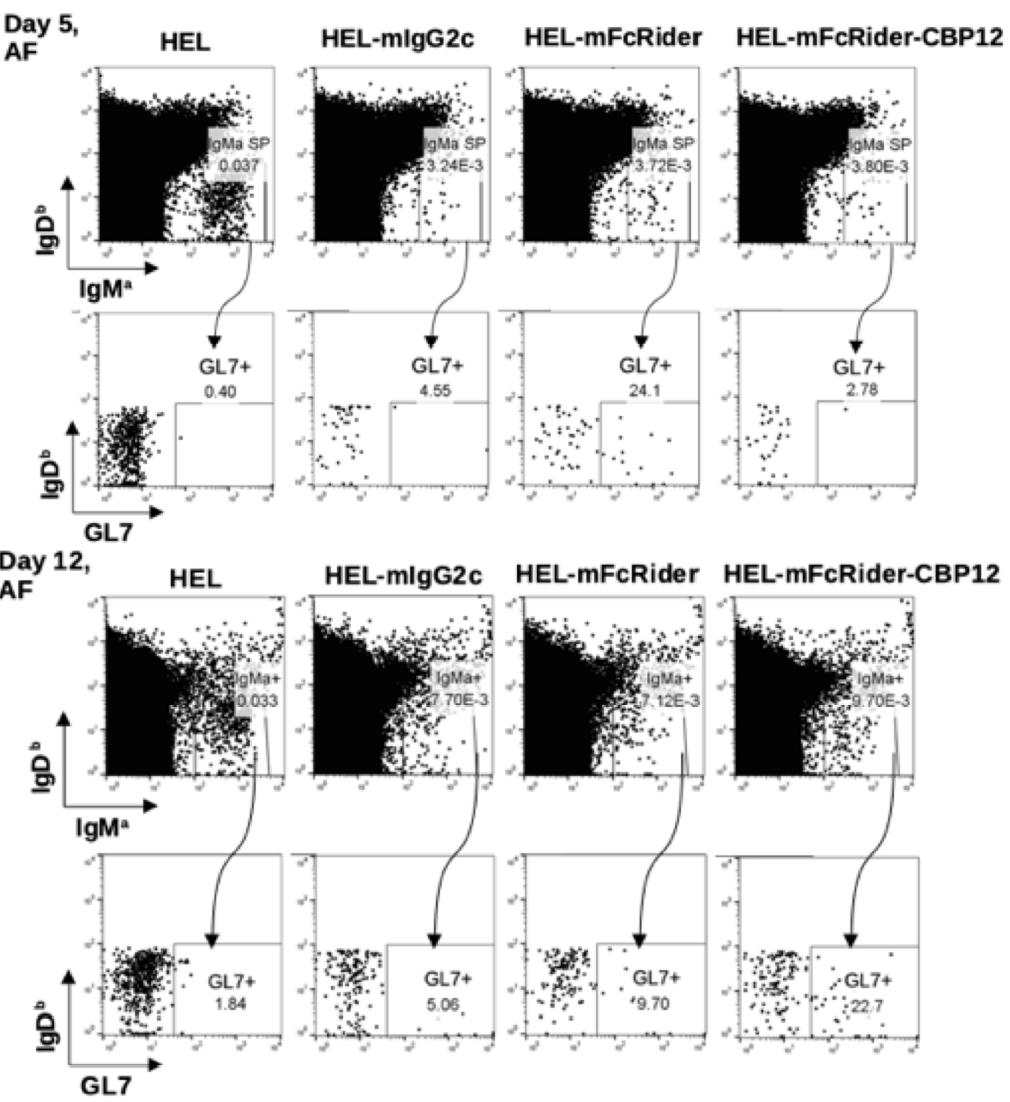


**Supplementary Fig. S3.** Oligomerized FcRider but not Fc has adjuvant effect and induces GL7^+^ GC B cells when presenting Ag HEL in anti-HEL BCR transgenic line MD4 (JAX, #002595, C57BL/6-Tg(IghelMD4)4Ccg/J). 3x10^6^ splenocytes from MD4 mice were transferred into syngeneic C57BL/6 mice, and one day later the recipients were immunized (i.p.) with commercial HEL protein (Sigma) without any fusion tag, or with recombinant HEL antigens in fusion with either mIgG2c, mFcRider, or mFcRider-CBP12. The recombinant HEL-based immunogens were produced by Fut8-/- CHO cells, hence were afucosalylated. The amounts of the injected immuogens were normalized based on their molecular weights. Note that the donor transgenic B cells express the IgM^a^ allotype, while the B cells from normal C57BL/6 recipients express the IgD^b^/IgM^b^ allotype. After 4-18 days of immunization, IgD^b-^IgM^a+^GL7^+^ GC B cells were analyzed by FACS. As the CBP12 moiety fused at the C-terminus of mFcRider may alter the distribution dynamics of the immunogen, the kinetics of GL7^+^ cell induction is faster for HEL- mFcRider than for HEL-mFcRider-CBP12.


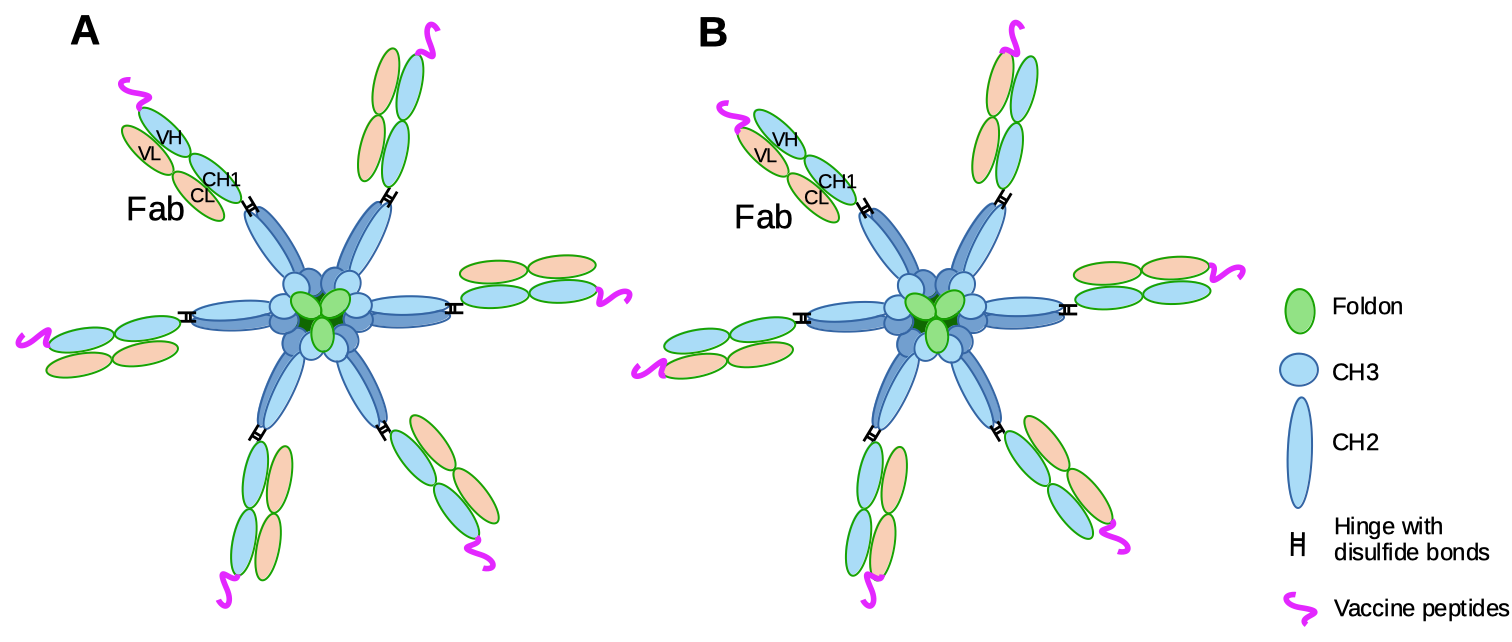
**Supplementary Fig. S4.** Illustration of Ag-Fab-FcRider that simultaneously functions as a neutralizing mAb and a self-adjuvanting vaccine in a single entity for “Hybrid Immunization”. The Fab portion of a neutralizing antibody can be fused at the N-terminus of the hexamer FcRider. Immunodominant subunits and/or antigenic B and T cell epitopes of a potential vaccine can be fused at the N-terminus of Fab, either H (A) or L chain (B), with flexible linkers. Note that the vaccine part should not be recognized by the Fab part to avoid the canceling effect. Ideally, the mAb and vaccine moieties should each aim for different targets to maximize the potential of “Hybrid Immunization”.

**Supplementary Table 1.** Immunoprofiling data of VH gene usage and HCDR3 length 20 among the three groups of UFO-BG, UFO-BG+AddaVax and UFO-BG+mFcRider AF.


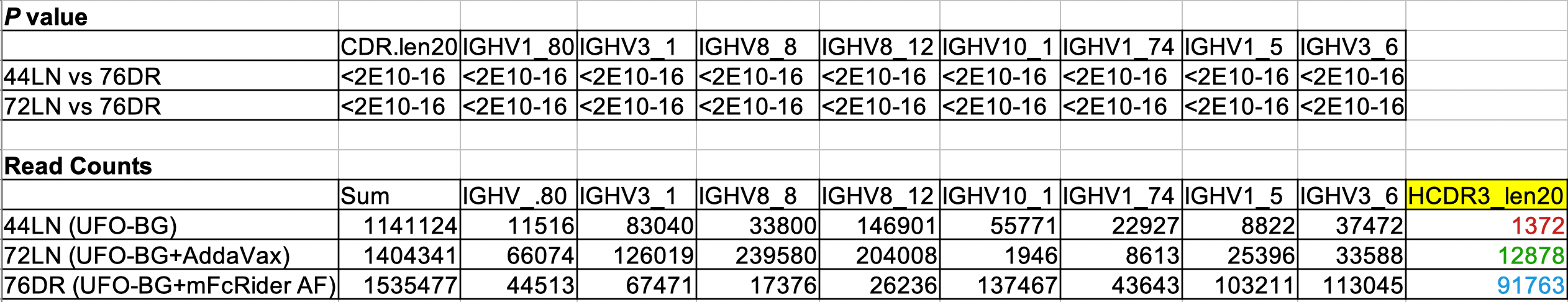

Supplement: Supplementary_Materials_tbae023 [file supplementary_materials_tbae023.docx]
